# Will the Public's Health Fall Victim to the Home Foreclosure Epidemic?

**DOI:** 10.1371/journal.pmed.1000087

**Published:** 2009-06-16

**Authors:** Gary G. Bennett, Melissa Scharoun-Lee, Reginald Tucker-Seeley

**Affiliations:** 1 Department of Psychology and Neuroscience, Duke University, Durham, North Carolina, United States of America; 2 Duke Global Health Institute, Duke University, Durham, North Carolina, United States of America; 3 Department of Society, Human Development & Health, Harvard School of Public Health, Boston, Massachusetts, United States of America; 4 Center for Community-Based Research, Dana Farber Cancer Institute, Boston, Massachusetts, United States of America

## Abstract

Gary Bennett and colleagues discuss the ways in which the dramatic rise in home foreclosures, particularly in the US, may have health consequences.

**Summary Points**

- While policy makers worldwide have scrambled to counter its economic effects, the potential health implications of home foreclosure have received little empirical attention.
- Home foreclosure can be viewed as a stressful life event of prolonged duration, with multiple phases of variable intensity.
- Although no studies to date have reported the specific health effects of home foreclosure, we posit that foreclosure may be associated with a range of psychological and health behavior outcomes that, in turn, might increase chronic disease risk.
- Susceptibility to home foreclosure might involve both compositional and contextual dimensions.
- Delinquency management policies designed to prevent foreclosures from occurring are arguably best suited to protect the health of those at greatest risk.

## Introduction

Over two million United States households have been affected by home foreclosure (or “home repossession”) in 2008 alone [1], and the epidemic shows no signs of abating. Following market downturns caused by the bursting of the dot-com bubble, mortgage interest rates were sharply lowered in the US and abroad [2],[3]. These actions resulted in massive home refinancing, dramatic increases in home demand, and higher home prices—from 1997–2006, home prices increased 124% in the US, with greater increases in Europe: 194% in Britain and 180% in Spain, and 253% in Ireland. Home ownership in the US also hit record levels [2],[3],[4] and was especially pronounced among racial/ethnic minorities, those of low socioeconomic status, and young adults [2], likely driven by the widespread availability of subprime mortgages [4],[5]. However, housing supply soon outstripped demand, prices dropped, and many homeowners—more than 7.5 million in 2005—owed more than their homes were worth [6]. Between 2006 and 2008, foreclosure filings increased 225% in the US [1].

While the literature has not fully explicated the health effects of foreclosure, related exposures have been linked with increased risk for several mental and physical health conditions [7]–[13]. This, combined with the frequent finding that home ownership has largely positive associations with health and well-being [14]–[17], suggests that the current raft of home foreclosures may represent an increasing health threat.

## Home Foreclosure as a Stressor

Losing a home through foreclosure is not a single occurrence. In the US, it is an often-protracted and highly aversive process, usually beginning with mortgage delinquency, which may lead the lender to initiate the legal process of foreclosure, which, if unresolved, can result in homeowner eviction and repossession of the home. Home sale proceeds are wholly retained by US banks, but in the United Kingdom and other nations, net profits (after debts are settled) are generally returned to homeowners. In the US, the foreclosure process differs substantially depending on the regulatory environment [18],[19], may include judicial supervision [20], and can range from several months to over a year [21],[22]. Thus, home foreclosure can be viewed as a stressful life event of prolonged duration, with multiple phases of variable intensity [23]. Indeed, several life event inventories [24],[25] and semi-structured interviews [26] have included foreclosure among the range of assessed events. For example, on the widely used Social Readjustment Rating Scale [24], which rates the stressfulness of 43 life events, foreclosure was originally rated number 21 in 1967. In a 1997 update, however, foreclosure surged to number 11 [27]. Interpreting the available evidence is challenging because the analytic convention has been to sum items on life events inventories [28], rather than to examine discrete events, so we know little about the independent effects of foreclosure. However, we suspect that if foreclosure-related stress surpasses one's ability to cope [29], it may unduly affect psychological functioning and health behavior practices—important health endpoints as well as tightly interrelated mechanisms through which foreclosure may heighten risk for several chronic conditions (e.g., cardiovascular disease) [30].

## Potential Psychological Responses to Home Foreclosure

The experience of stressful life events has been implicated in the etiology of both anxiety and depressive disorders [31]–[33]. There is particularly strong evidence that stressful life events are causally related with the initial episode of depression. The intensity of the foreclosure process may make it especially deleterious, as depression risk increases in a dose-response manner [34] with the severity and number of stressful life events experienced. Several additional issues are particularly concerning with respect to depression outcomes. First, it appears that chronic stressors (e.g., job strain, financial strain) can exacerbate the impact of adverse life events on depression outcomes, particularly when the domain of the two exposures is concordant. This is salient because home foreclosure typically occurs amidst long-term financial difficulties, and thus may be tied to chronic stressors that have known associations with adverse health outcomes [7]–[13]. For example, chronic financial strain has been positively associated with depressive symptomatology in populations from the US [35]–[38], UK [11], and China [39]. Next, relative to fateful occurrences, depression is more strongly related to stressful life events for which the individual perceives having some responsibility [34]. This belief might be particularly common among the foreclosed [40], despite widespread acknowledgment of deceptive mortgage industry practices [41]–[43]. Additionally, concern regarding one's limited personal control of the foreclosure process may also intensify the impact of stress on depression [44],[45]. Several studies have shown that depressed individuals can act in ways that promote their subsequent exposure to additional negative life events (e.g., occupational problems, financial difficulties, interpersonal conflict) [46]. Together, there appears to be potential for the already daunting global burden of depression [47] (including its role as a risk factor for cardiovascular disease [48]) to be magnified by the foreclosure crisis.

## Potential Impact of Home Foreclosure on Health Behaviors

Unhealthful behaviors may be used to cope with stressful life events. Stress is positively associated with myriad such health behaviors, including tobacco use [49],[50], alcohol consumption [51], sleep dysregulation [52], and weight gain [53]–[56]—perhaps via decreased physical activity [56] and increased consumption of energy-dense foods [57]–[59]. Home foreclosure may also impact health care utilization. Financially stressed individuals report fewer preventive doctor visits [60],[61] and reduced prescription medication adherence [62]. If current economic trends continue, this may become increasingly common; a recent survey showed that given the economic climate over half of Americans aged 45 and older switched to generic or non-prescription drugs, 16% delayed preventive care, and over one-fifth delayed seeing a doctor [63].

## Who Might Be Most Vulnerable to the Health Effects of Home Foreclosure?

Susceptibility to home foreclosure might involve both compositional and contextual dimensions. Those with lower socioeconomic status and some ethnic minorities may have higher likelihood and severity of exposure, as well as potential challenges in securing stress-buffering resources. At the individual level, most of those who experience foreclosures will not exhibit adverse health effects [64]. Even in the face of extreme stressors, most people are sufficiently resilient to stressful events [65]. However, individual characteristics such as prior psychiatric or adverse health histories [30], poor coping skills [35],[66]–[68], low social support [35], neuroticism [69], low self-esteem [70], and highly valuing economic success [71] may heighten vulnerability.

The macroeconomic context has had profound and far-ranging effects that might exacerbate foreclosure's potential health effects. Unemployment in developed nations is at historic levels, and home prices show no immediate signs of rebounding. Soaring food, energy, and health care prices in recent years have added to the financial strain of the average household [2]. Whether the macroeconomic climate directly impacts individual health is disputed [72]–[75], but adverse contextual circumstances are more prevalent in times of economic decline and may interact with foreclosure to increase stress exposure. Among the range of problematic macroeconomic indicators [72]–[75], unemployment is arguably most concerning [7],[76]–[78]. In better economic cycles, opportunities to mitigate the ill effects of job loss (e.g., re-employment, loan refinancing, social services) may be more plentiful. However, the combination of unemployment (which itself poses health risks [79]–[82]) and foreclosure in the current economic environment may be particularly deleterious.

When foreclosures occur, they are accompanied by significant externalities at the neighborhood level [83]–[85] that might impact resident's health. For example, foreclosures spur neighborhood disinvestment, home vacancies, and property abandonment [18], which can result in lower property values, reduced local services [22], and violent crime [86]. When foreclosures reach a critical mass [22], these varied problems can economically weaken the neighborhood [87] and create a sense of social disorder, fear, and distrust [88],[89], all of which may negatively influence residents' health and health behaviors [90],[91]. Neighborhoods hardest hit by the foreclosure crisis (older, urban areas [21],[92]) were previously improving in stability [19],[93]; however, continuous foreclosures in these neighborhoods may threaten stability and decrease resident social capital, which might in turn heighten associated health risks [94],[95].

## Priorities for Future Research

As noted, we are unaware of any studies that have specifically investigated the health effects of home foreclosure. In a closely related report, however, Taylor [96] recently showed that UK residents with housing payment problems had poorer levels of psychological well-being, independent of financial hardship. These psychological costs were positively related to financial problems of greater intensity and duration. Several questions emerge from this and other work. First, how does home foreclosure interact with other stressful life events (e.g., job loss, medical costs [79],[97],[98]) and/or chronic stressors (e.g., financial strain [11],[99]) to impact health outcomes? Among the chronically stressed (e.g., those in persistent poverty), is a saturation effect observed, i.e., are such individuals more resilient to the stress of home foreclosure [100]? Also, it is unclear how the macroeconomic climate might exacerbate, or even inoculate (given the increasingly normative nature of foreclosure) individuals to foreclosure stress. Research is necessary to examine how home foreclosure impacts other household members, such as partners and dependent children [7]. Finally, given the social patterning of mortgage lending [44],[101],[102], future studies should examine whether widening of racial/ethnic and socioeconomic disparities in foreclosure-related health outcomes has occurred. Widespread variation in foreclosure exposure affords the unfortunate opportunity to study these and other questions using “natural experiment” investigations.

## Policy Approaches

Two broad categories of home foreclosure remediation policies have received most attention: (1) those that prevent the onset of the foreclosure process, and (2) those that delay home eviction following mortgage delinquency. In the US for example, several states have passed legislation to help homeowners prevent foreclosures by increasing mortgage industry oversight, improving loan term disclosures, and requiring lenders to formally communicate with borrowers prior to foreclosure initiation. The Obama administration has introduced the Homeowner Affordability and Stability Plan, which allows for easier mortgage refinancing for non-delinquent homeowners. This is in contrast to a policy proposed by the former Bush administration (and endorsed by Obama as a candidate) that would have instituted national foreclosure moratoria, allowing more time for renegotiation of loan terms prior to eviction.

As foreclosure prevention policies have been debated over the past 18 months, health has been infrequently mentioned—perhaps understandable given the limited evidence of foreclosure's health effects. Nevertheless, delinquency management policies designed to prevent foreclosures from occurring are arguably best suited to protect the health of those at greatest risk. Even though overextended homeowners' chronic financial strain would likely continue, early intervention policies would ensure that individuals are protected from the exacerbating effects of foreclosure-related stress. This is in contrast to policies that extend the period of foreclosure preceding eviction. Although the individual and neighborhood benefits of such policies are not trivial, they also have the potential to transform home foreclosure into a chronic stressor, which could magnify stress exposure and health risks.

It is particularly challenging to determine which policies are most beneficial to the health of delinquent homeowners facing imminent eviction. Such policies are likely to vary considerably across nations, given differences in the magnitude of exposure as well as variation in societal perspectives regarding the provision of assistance for what may be perceived to be a “personal responsibility.” In the US for example, it seems unlikely that foreclosure prevention policies will be enacted with the specific goal of offsetting foreclosure-associated health risks, but other nations offer useful models. For example, the UK has recently announced a plan to facilitate referrals for psychological counseling to assist those facing unemployment and debt, including the scores affected by housing repossession [103]. Additionally, strategies that would assist families to identify permanent, affordable housing might ease their residential transition following foreclosure.

## Conclusion

Although current foreclosure rates are unprecedented, such economic downturns are generally thought to be cyclical, suggesting that recovery may be on the horizon [2]. However, the near-term outlook for many homeowners is poor, as home prices are expected to continue to decline. A recent United Nations report projected that 50 million job losses will occur worldwide, which will likely magnify the current foreclosure crisis [104]. Successful governmental responses to the foreclosure crisis specifically, and to the global economic crisis in general, will require health and social policy coordination that safeguards household income, stabilizes commodity prices, helps citizens maintain health care, and prevents disruption in children's education [105]. In so doing, short- and long-term health effects of the foreclosure epidemic might be mitigated.
